# Choice of reaction progress variable under preferential diffusion effects in turbulent syngas combustion based on detailed chemistry direct numerical simulations

**DOI:** 10.1038/s41598-024-64552-0

**Published:** 2024-06-27

**Authors:** Vinzenz Silvester Wehrmann, Nilanjan Chakraborty, Markus Klein, Josef Hasslberger

**Affiliations:** 1 Department of Aerospace Engineering, University of the Bundeswehr Munich, Werner-Heisenberg-Weg 39, 85577 Neubiberg, Germany; 2 School of Engineering, Newcastle University, Claremont Road, Newcastle-Upon-Tyne, NE1 7RU UK

**Keywords:** Syngas combustion, Turbulent premixed combustion, Reaction progress variable, Hydrogen combustion, Direct numerical simulation, Applied mathematics, Fluid dynamics

## Abstract

The combustion of hydrogen and carbon-monoxide mixtures, so-called syngas, plays an increasingly important role in the safety context of non-fossil energy generation, more specifically in the risk management of incidents in process engineering plants for ammonia synthesis and in nuclear power plants. In order to characterize and simulate syngas/air combustion on industrially relevant scales, subgrid modelling is required, which is often based on a reaction progress variable. To understand the influence of different fuel compositions, turbulence intensities and flame topologies on different possible definitions of reaction progress variable, detailed chemistry direct numerical simulations data of premixed, lean hydrogen/air and syngas/air flames has been considered. A reaction progress variable based on normalized molecular oxygen mass fraction has been found not to capture the augmentation of the normalized burning rate per unit flame surface area in comparison to the corresponding 1D unstretched premixed flame due to preferential diffusion effects. By contrast, reaction progress variables based on other individual species, such as hydrogen, can capture the augmentation of the rate of burning well, but exhibit a pronounced sensitivity to preferential diffusion effects, especially in response to flame curvatures. However, a reaction progress variable based on the linear combination of the main products can accurately represent the temperature evolution of the flame for different mixtures, turbulence intensities and varying local flame topology, while effectively capturing the augmentation of burning rate due to preferential diffusion effects. However, its tendency to assume values larger than 1.0 in the regions of super-adiabatic temperatures poses challenges for future modeling approaches, whereas the reaction progress variable based on hydrogen mass fraction remains bound between 0.0 and 1.0 despite showing deviations in comparison to corresponding variations obtained from the unstretched laminar flame depending on flame curvature variations.

## Introduction

Synthesis gas or syngas, a combination of hydrogen and carbon-monoxide, plays a crucial role in process engineering. It is a key component in various industrial processes, including the Fischer–Tropsch process for gas-to-liquids conversion^1^ and the production of ammonia using the Haber-Bosch process^2^. With ammonia gaining prominence as a suggested fuel to decarbonize combustion processes^3^, syngas produced with renewable energy sources^4^ is consequently gaining importance as well and might even be considered as a possible fuel for carbon-neutral combustion processes^5,6^. Since the processing of syngas is often conducted under elevated pressure^7^, it poses potential safety risks in terms of gas leakage and the resulting formation of flammable mixtures in (contained) atmospheres. Furthermore, syngas represents a relevant safety aspect in the non-fossil generation of electrical energy, as it is produced in the event of a meltdown accident in nuclear power plants through the interaction of molten corium with the concrete of the reactor building, and subsequently released into the containment^8^.

In the context of safety and risk management for processing facilities and nuclear power plants, it is imperative to characterize and simulate the premixed combustion process of syngas/air mixtures on industrially relevant spatial scales. To achieve this, subgrid modeling approaches must be developed. These approaches often rely on reaction progress variables to describe the combustion process. Especially for lean, premixed, turbulent and hydrogen-rich combustion processes, the capability to capture accelerated flame behavior, induced by intrinsic flame instabilities, is required for evaluations based on the reaction progress. The seminal work by Darrieus^9^ and Landau^10^ on the destabilizing impact of thermal expansion concerning small flame perturbations, quantified in terms of $\sigma = \rho _b / \rho _u$ with $\rho$ being the gas density and subscripts *b* and *u* denoting the burned and unburned values, did not consider diffusive effects. Barenblatt, Zel’Dovich, and Istratov investigated these thermo-diffusive effects^11^, indicating that mixtures containing deficient reactants with Lewis numbers $Le = D_\alpha / D_i$, where $D_\alpha$ signifies the mixture’s thermal diffusivity and $D_i$ denotes the mass diffusivity of the considered species, notably below unity (typically obtained for hydrogen) tend to develop thermo-diffusive instabilities. In such cases, the higher diffusivity of the deficient reactant compared to the mixture’s thermal diffusivity results in an increased chemical reaction rate at convex flame fingers that extend into the unburned gas, while simultaneously reducing the reaction rate at concave flame regions, amplifying the development of convex flame fingers into the unburned gas. This characteristic behavior has to be captured by modelling methodologies which often employ a reaction progress variable for characterizing the thermo-chemical state. Furthermore, the species profiles in detailed chemistry simulations tend to be influenced by preferential diffusion effects, particularly evident for curved flame elements^12^. Given the notable influence of the choice of reaction progress variable on modeling approaches^13^, evaluating reaction progress with minimal sensitivity to local flame topology is crucial. Several previous analyses^14–18^ considered different choices of reaction progress variables based on normalized mass fractions of $\mathrm {O_2}$, major species, fuel and product mass fractions and non-dimensional temperature for hydrocarbon fuels where the effects of preferential diffusion of heat and species are not strong. While several studies have already dealt with this aspect for compound fuel mixtures consisting of hydrogen and hydrocarbons^19–26^, it has rarely been addressed in the existing literature for syngas where the effect of preferential diffusion plays a key role, and the current analysis fills this gap. The present work analyses the effects of preferential diffusion on the normalized species profiles within the flame front in syngas-air premixed flames under different mixture compositions and turbulence intensities.

In this respect, it is worthwhile to identify a reaction progress variable based on the profiles of major species mass fractions so that it accurately characterizes the lean, premixed, turbulent combustion process for a wide range of different hydrogen/air and syngas/air mixtures, including variations in fuel composition and equivalence ratio, and different flame-turbulence interaction regimes. To accomplish this goal, a Direct Numerical Simulation (DNS) study based on a detailed chemistry approach is conducted. The simulation results are analyzed to assess the impact of preferential diffusion and intrinsic flame instabilities on various choices of reaction progress variables.

## Mathematical background

The reaction progress variable mathematically normalizes the progress of the combustion process, ranging from zero (representing the unburned gas mixture) to one (indicating the fully burned state of the gas mixture). On the one hand, this progression can also be expressed using a temperature-based reaction progress variable, defined as follows:

1 $$\begin{aligned} c_\Theta = \left( T - T_{u,L}\right) / \left( T_{b,L} - T_{u,L} \right) , \end{aligned}$$

where $c_\Theta$ denotes the normalized temperature and $T_u$, $T_b$, and *T* represent the unburned gas temperature, the adiabatic flame temperature of the unstretched 1D adiabatic laminar premixed flame with the same mixture composition, and the instantaneous dimensional temperature, respectively. The subscript *L* corresponds to the values of a respective laminar flame. On the other hand, the progress of chemical activities can be assessed based on chemical species using a mass fraction-based reaction progress variable:

2 $$\begin{aligned} c_i = \left( Y_{i,u,L} - Y_i\right) / \left( Y_{i,u,L} - Y_{i,b,L}\right) , \end{aligned}$$

where $Y_i$ represents the mass fraction of species *i* and the subscripts *u*, *L* and *b*, *L* refer to the values associated with the unburned and burned gas properties in a 1D adiabatic unstretched laminar premixed flame for the same mixture composition, respectively.

Since the DNS study incorporates detailed chemistry, it offers the opportunity to analyze not only the evolution of mass fractions corresponding to fuel, oxidizer and products but also to assess the reaction progress of individual chemical species and linear combinations thereof. The mass fractions of combining all fuel species as well as all major products are defined as^27^:

3 $$\begin{aligned} Y_f&= Y_{H_2} + Y_{CO} \end{aligned}$$

4 $$\begin{aligned} Y_p&= Y_{H_2O} + Y_{CO_2}. \end{aligned}$$

To interpret the results of the DNS study and account for the typically non-unity Lewis numbers of the reactants in lean, hydrogen-based combustion processes, Joulin and Mitani^28^ introduced an effective Lewis number, defined as:

5 $$\begin{aligned} Le_{eff} = 1 + \frac{(Le_{O_2} - 1) + (Le_f -1)\mathscr {A}}{1 + \mathscr {A}}, \end{aligned}$$

where $\mathscr {A}$ details the influence of the equivalence ratio $\phi$ on the effective Lewis number of lean mixtures.

6 $$\begin{aligned} \mathscr {A} = 1 + \beta \left( \frac{1}{\phi } -1\right) . \end{aligned}$$

A solely temperature based approach^29^ is used to describe the Zel’Dovich number, resulting in

7 $$\begin{aligned} \beta = \frac{T_{b,L} - T_{u,L}}{T_{b,L} - T_{in}}, \end{aligned}$$

with the flame’s inner layer temperature $T_{in}$. Attili et al.^30^ described the inner layer temperature as the flame’s temperature value at the inflection point of the corresponding laminar flame profile $T_{in} = T_{L, \max \nabla T}$.

Due to multi-fuel mixtures being at the scope of this work, the Lewis numbers of the two fuels are combined through a molar-weighted approach as proposed by Dinkelacker et al.^31^

8 $$\begin{aligned} Le_f = \frac{1}{\frac{1-\alpha }{Le_{H_2}}+\frac{\alpha }{Le_{CO}}}, \end{aligned}$$

where $\alpha$ denotes the molar percentage of carbon-monoxide in the fuel.

## Numerical method and simulation setup

In this study, simulations of statistically planar, premixed turbulent flames are conducted using the DNS code SENGA2^32^. The simulations encompass different scenarios, including pure hydrogen/air flames and hydrogen/carbon-monoxide/air flames. SENGA2 solves the three-dimensional, fully compressible Navier-Stokes equations for reacting flows. The spatial discretization is achieved using a 10th-order central-difference scheme, where the accuracy drops progressively to a 4th-order one sided scheme for non-periodic boundaries. The explicit time advancement is accomplished through a fourth-order, low-storage Runge-Kutta method^33^. The computational domain is structured as a cuboid, discretized by a uniform Cartesian grid consisting of $1024 \times 512 \times 512$ equidistant points. The spacing between each discretization point is set to $\Delta x,y,z = 3.0 \times 10^{-5} m$. This resolution ensures at least 13 points per thermal flame thickness, which is defined as:

9 $$\begin{aligned} \delta _{th} = (T_{b,L} - T_{u,L}) / \max |\nabla T|_L. \end{aligned}$$

**Figure 1:**
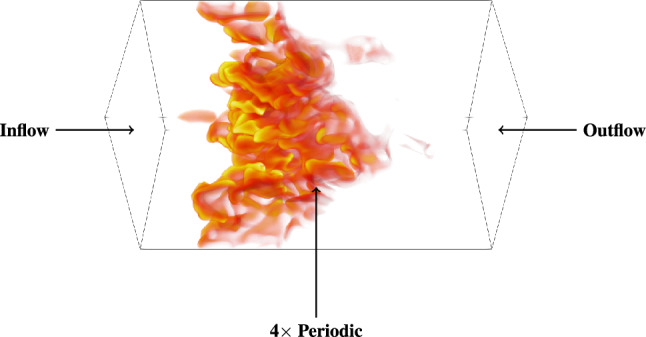
Schematic sketch to visualize the computational domain, together with the boundary conditions. The flame is illustrated via a volume rendering of the atomic hydrogen mass fraction.

A pure hydrogen-air flame with an equivalence ratio $\phi = 0.5$, representing the extreme case of syngas/air combustion without any carbon-monoxide in the fuel blend, is considered to be the reference case. The computational domain is taken to be a rectangular cuboid with inflow, featuring the initial conditions $T_0 = 293.15 \, K, p_0 = 1013.25 \, hPa$, and outflow boundaries in the direction of mean flame propagation for statistically planar flames considered here. The transverse boundaries are taken to be periodic. All boundary conditions are specified in accordance with the Navier-Stokes Characteristic Boundary Condition (NSCBC) formalism^34^. The spatial dimensions of the computational box are $\sim66\, \delta _{th,ref} \times 33\, \delta _{th,ref} \times 33\, \delta _{th,ref}$, where at least 15 grid points resolve $\delta _{th,ref}$. A schematic sketch of the computational domain, based on the $\phi = 0.6, \alpha = 25\%$ case, is shown in Fig. 1.

SENGA2 implements simulations with detailed multi-step chemistry and detailed multi-species diffusion. In this work, a mixture-averaged transport approach is used, which assembles transport data for each individual species. To describe the chemistry, an optimized kinetic model for the combustion of hydrogen and carbon-monoxide based on the work by Davis et al.^35^ is implemented. This mechanism encompasses 14 chemical species and 34 elementary reactions. The initialization of the 3D flame is accomplished by imposing a laminar flame profile derived from previously conducted one-dimensional simulations. Additionally, turbulence is imposed using decaying turbulent flow fields with various turbulence intensities $u^\prime /S_{L}$ as well as a normalized integral length scale of $L_{11} / \delta _{th,ref} = 3.5$.

**Table 1:** Attributes of DNS cases considered here: equivalence ratio $\phi$, ratio of mole fractions of CO and H$_2$ in the fuel-blend $\alpha$, mole fractions of hydrogen and carbon-monoxide (i.e. $X_{H_2}$ and $X_{CO}$) in the unburned gas, normalized turbulence intensity $u^\prime /S_L$ and their respective effective Lewis number $Le_{eff}$ (see Eq. 5).

$\phi$	$\alpha$ (%)	$X_{H_2}$ (%)	$X_{CO}$ (%)	$u^\prime /S_L$	$Le_{eff}$
**0.5**	**0**	**17.36**	**0**	**4.0**	**0.587**
0.5	25	13.02	4.340	4.0	0.615
0.5	25	13.02	4.340	6.551	0.615
0.6	0	20.13	0	4.0	0.674
0.6	25	15.10	5.033	4.0	0.690
0.6	50	10.06	10.06	4.0	0.728
0.717	25	17.36	5.787	2.358	0.768
0.717	25	17.36	5.787	4.0	0.768

The reference case is highlighted in bold.

The DNS study considers various syngas mixtures, as detailed in Table 1. The chosen cases are intended to investigate the impact of the addition of carbon-monoxide and the partial substitution of hydrogen by carbon-monoxide on the hydrogen/air combustion process, namely on the influence of preferential diffusion and the possibilities of intrinsic instabilities and eventually on the choice of reaction progress variable. In accident scenarios, the release of hydrogen as well as carbon-monoxide may reflect a dynamic progression. In the context of a nuclear power plant meltdown scenario, hydrogen is emitted initially into the containment atmosphere, whereas carbon monoxide generation occurs in subsequent stages, promoting the potential ignition of both pure hydrogen/air and diverse syngas/air mixtures. Hence, the cases are selected to represent either a constant equivalence ratio $\phi$, but for different extents of substitution by carbon-monoxide in the unburned hydrogen/air mixture or a fixed molar fraction value for hydrogen $X_{H_2}$ with the addition of carbon-monoxide in the unburned gas mixture, compared to their respective pure hydrogen/air flame. Furthermore, in two cases the turbulence intensity is varied to values deviating from $u^\prime / S_L = 4$ to determine the influence of the effects of preferential diffusion under different turbulence intensities. With Karlovitz numbers $Ka = \left( L_{11}/\delta _{th}\right) ^{-0.5} \left( u' / S_L \right) ^{1.5}$ in the range of $Ka \approx 1\dots 9$ and turbulence intensities in the range of $u^\prime / S_L \approx 2\dots 7$, all considered cases are classified in the thin reaction zones regime. The simulations are conducted over a simulation duration of three eddy turnover times, as defined by:

10 $$\begin{aligned} \tau _{et}=L_{11}/u^\prime . \end{aligned}$$

## Results and discussion

**Figure 2:**
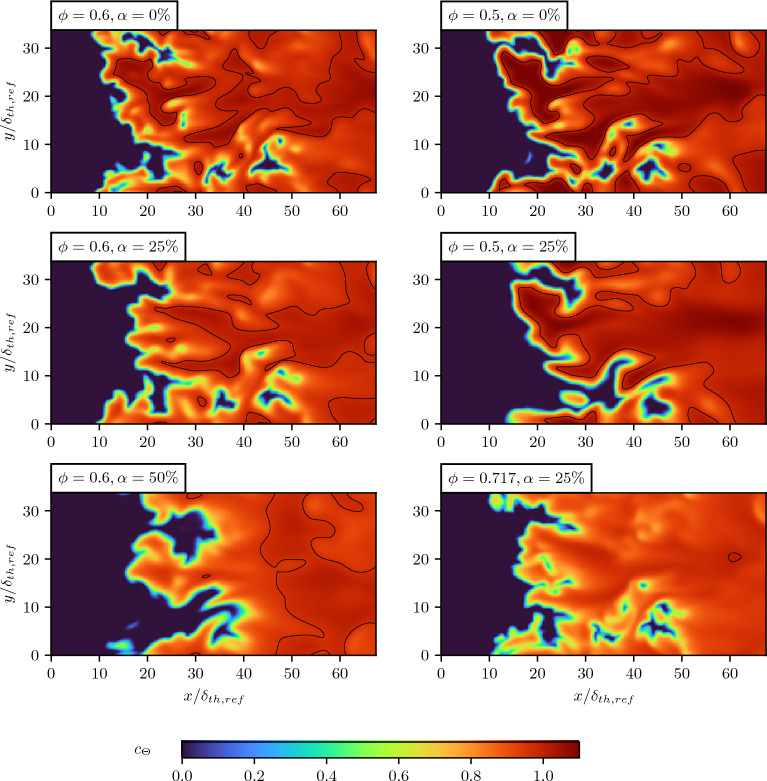
Distribution of the instantaneous normalized temperature $c_\Theta$ in the $x-y$ midplane for all $u^\prime / S_L = 4.0$ cases at $\tau _{et}\approx 3$. Additionally, the iso-contour for $c_\Theta =1.0$ (black) encloses the super-adiabatic temperature (i.e., $c_\Theta >1.0$) regions.

The distributions of the instantaneous, non-dimensional temperature $c_\Theta$ at the $x-y$ midplane are shown in Fig. 2 for all $u'/S_L=4.0$ cases considered here. In all presented cases, pronounced wrinkling is observed, along with the presence of super-adiabatic temperature (i.e., $c_\Theta >1.0$) regions. To accentuate these super-adiabatic regions, an iso-contour is plotted at the value $c_\Theta = 1.0$.

Since the normalized turbulent flame area serves as an indicator of flame wrinkling, a growth of volume-integrated burning rate at the same rate implies that the increased burning rate results solely from the augmented flame surface area. This is in accordance with the expectations of Damköhler’s first hypothesis^36,37^, where a larger flame area contributes to an enhanced volume-integrated burning rate. By contrast, non-identical evolutions of volume-integrated burning rate and flame surface area under negligible flame stretch are indicative of considerable preferential diffusion effects, which impact the consumption of the involved chemical species. To quantify this ratio, the reaction rate of the reaction progress variable per unit area of the turbulent flame normalized by the corresponding value under laminar conditions is assessed. This is defined as:

11 $$\begin{aligned} \Omega _i = \left( S_{T,i} A_\perp \right) / \left( S_{L} A_{T,i} \right) , \end{aligned}$$

with values of $\Omega > 1$ typically observed in statistically planar flames with effective Lewis numbers significantly smaller than unity^28^. The turbulent burning velocity is determined through the associated chemical reaction rate:

12 $$\begin{aligned} S_{T,i} = \frac{\smallint \dot{\omega _i} \, dV }{\rho _u A_\perp \left( Y_{b,i} - Y_{u,i} \right) }, \end{aligned}$$

whereas the turbulent flame area is determined by integrating the surface density function of the relevant reaction progress variable^38^:

13 $$\begin{aligned} A_{T,i} = \smallint |\nabla c_i| \, dV. \end{aligned}$$

The cross-sectional area of the domain normal to the *x*-direction is given by $A_\perp$. The chemical reaction rates for the linear combination cases are obtained similarly to the combination of mass fractions:

14 $$\begin{aligned} \dot{\omega }_f&= \dot{\omega }_{H_2} + \dot{\omega }_{CO} \end{aligned}$$

15 $$\begin{aligned} \dot{\omega }_p&= \dot{\omega }_{H_2O} + \dot{\omega }_{CO_2}. \end{aligned}$$

The normalized burning rate per unit area of the flame is assessed in the quasi-steady state ($\tau _{et} > 2.5$) of the temporal evolution of both the turbulent burning velocity and flame surface area for all cases included in the DNS study, as shown in Table 2. It is evident that the hydrogen-based, the fuel-based and the product-based evaluations of $\Omega$ result exclusively in values above unity, indicating the influence of preferential diffusion effects characterized by an effective Lewis number smaller than unity. This is consistent with the super-adiabatic temperature spots (i.e., $c_\Theta >1.0$), as visible in Fig. 2 where all cases, with the exception of the case $\phi =0.717$, show notable super-adiabatic regions. However, the oxygen-based reaction progress variable exhibits $\Omega _{O_2} < 1$ for all cases, which is caused by an elevated oxygen-based turbulent flame area $A_{T,O_2}$. This opposite behavior of $\Omega _{O_2} < 1$ compared to $\Omega>1$ for the other considered definitions makes the choice of reaction progress variable a challenging task for these flames.

**Table 2:** Normalized burning rate per unit area of the flame $\Omega$, evaluated based on hydrogen, oxygen as well as the sum of fuel species and major products, at $\tau _{et} \approx 3$ for the cases considered in the DNS study.

$\phi$	$\alpha$ (%)	$u^\prime /S_L$	$\Omega _{H_2}$	$\Omega _{O_2}$	$\Omega _f$	$\Omega _p$
**0.5**	**0**	**4.0**	**1.845**	**0.867**	**1.845**	**1.234**
0.5	25	4.0	1.807	0.301	1.437	1.216
0.5	25	6.551	2.034	0.963	1.538	1.328
0.6	0	4.0	1.542	0.772	1.534	1.104
0.6	25	4.0	1.578	0.158	1.355	1.150
0.6	50	4.0	1.567	0.308	1.299	1.140
0.717	25	2.358	1.314	0.795	1.300	1.057
0.717	25	4.0	1.409	0.105	1.290	1.106

The reference case is highlighted in bold.

**Figure 3:**
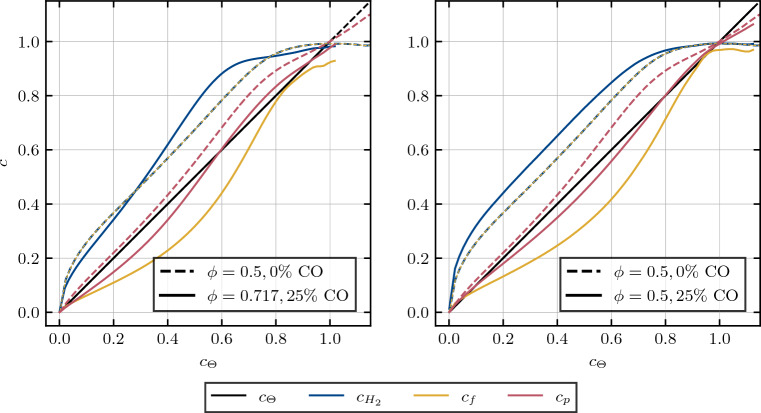
Profiles of mean values of reaction progress variables *c* conditioned upon non-dimensional temperature for the addition (left) and substitution (right) of/by carbon-monoxide for turbulence intensities of $u^\prime / S_L = 4.0$. Dashed lines represent the reference case of $\phi = 0.5$, while solid lines represent cases with $25\%$ molar carbon-monoxide in the fuel blend. Note that the dashed blue line and the dashed yellow line are lying on top of each other.

The influence of the addition and substitution of/by carbon-monoxide on the normalized species distribution in the form of reaction progress variables is presented in Fig. 3. The preferential diffusion contributes to the deviation of the reaction progress variable from the non-dimensional temperature. Starting with a pure hydrogen/air flame at an initial equivalence ratio of $\phi = 0.5$, the addition of carbon-monoxide results in an increased equivalence ratio of $\phi = 0.717$, corresponding to a molar fuel composition of $75\%$ hydrogen and $25\%$ carbon-monoxide and leading to an increase in the adiabatic flame temperature from $T_{b,\phi =0.5,\alpha =0\%} = 1636.63 \,K$ to $T_{b,\phi =0.717,\alpha =25\%}=2071.59 \, K$.

Considering the hydrogen-based reaction progress variable, the addition of carbon-monoxide results in a pronounced deviation from the corresponding profile in the reference case. Moreover, towards the burned side of the flame, as the temperature rises, the hydrogen based reaction progress variable increases and even surpasses the reaction progress value obtained in the reference case before reaching a plateau at $c_\Theta \approx 0.7$. An increase of the adiabatic flame temperature as a result of carbon-monoxide addition is indicative of the strengthening of the effects of thermal expansion and flame normal acceleration^9,10,39,40^. The corresponding elevated equivalence ratio acts to increase the effective Lewis number^28^ of the mixture to approach values closer to unity. Consequently, the effects of preferential diffusion weaken with an increase in equivalence ratio, which can be substantiated by Fig. 3 (left) and the near absence of super-adiabatic temperature regions for the $\phi = 0.717$ case (see Fig. 2).

A contrasting behavior is observed for the substitution by carbon-monoxide. While only mildly increasing the adiabatic flame temperature, reaching $T_{b, \phi =0,5,\alpha =25\%} = 1668.26K$, the molar percentage of hydrogen decreases from $X_{H_2} = 17.36\%$ to $X_{H_2} = 13.02\%$, subsequently increasing hydrogen’s deficiency as a reactant, which leads to an enhancement of preferential diffusion effects^41,42^, as can be seen from the variation of the hydrogen-based reaction progress variable profile in Fig. 3 (right). This behavior is consistent with the effects associated with sub-unity Lewis number such as the appearance of super-adiabatic temperature regions in Fig. 2. It can be seen from Fig. 3 that the fuel-based reaction progress variable $c_f$ is highly sensitive to both the addition and substitution of/by carbon-monoxide, and shows a strong non-linear dependence of temperature for both pure hydrogen/air and syngas/air cases and it has been found to be sensitive to the preferential diffusion effects within the flame. In contrast, the product-based reaction progress variable yields promising results, effectively capturing the temperature change within the flame in the least non-linear manner for both pure hydrogen/air and syngas/air cases while also remaining insensitive to both addition and substitution.

Various reaction progress variables are assessed in both the concave and convex regions of the flame to evaluate the influence of local flame topology on preferential diffusion effects and subsequently on reaction progress. To distinguish between these regions, the curvature of the flame is calculated based on the respective reaction progress variable $c_i$ as described^43^:

16 $$\begin{aligned} k_{m,i} = ( \nabla \cdot \mathbf {n_i}) / 2, \end{aligned}$$

considering the normal vector to the flame front, denoted as $\textbf{n}$, which is defined as:

17 $$\begin{aligned} \mathbf {n_i} = - \nabla c_i / |\nabla c_i|. \end{aligned}$$

**Figure 4:**
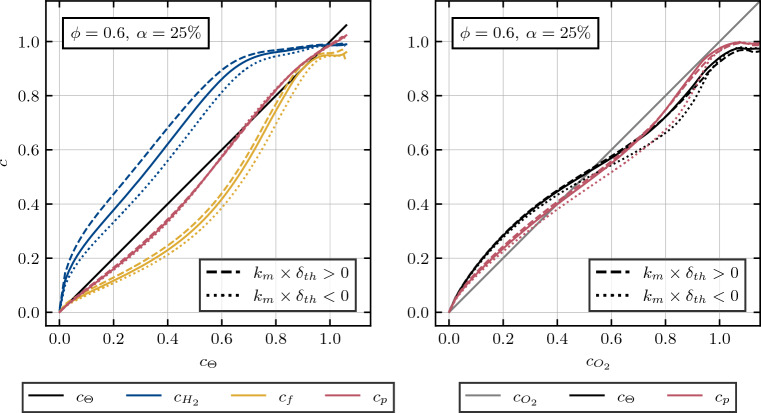
Profiles of mean values of reaction progress variables *c* conditioned upon non-dimensional temperature (left) and the reaction progress of oxygen (right) evaluated for different levels of flame curvature $k_{m,\Theta }$ at a turbulence intensity of $u^\prime / S_L = 4.0$. Dashed lines represent an evaluation conditional on mean curvature levels $k_{m,\Theta } \times \delta _{th}> 0$, dotted lines represent an evaluation conditional on $k_{m,\Theta } \times \delta _{th}< 0$ respectively, while the solid line represents an evaluation independent on curvature.

In this study, the normal vector and subsequently the mean curvature are evaluated in relation to the reaction progress variable associated with normalized temperature development. The convex flame fingers are associated with $k_{m,\Theta } \times \delta _{th} > 0$, while concave regions are characterized by values below $k_{m,\Theta } \times \delta _{th} < 0$, as illustrated in Fig. 4 for an equivalence ratio $\phi = 0.6$ and the fuel’s molar amount of carbon-monoxide $\alpha = 25\%$. The reaction progress of hydrogen, in accordance with the previously discussed preferential diffusion effects, is significantly impacted by flame curvature values. Convex flame fingers exhibit elevated reactivity towards the unburned side of the flame, while concave regions experience reduced reactivity. In contrast, carbon-monoxide remains unaffected by curvature variations towards the unburned side of the flame. However, towards the burned side of the flame, the elevated temperatures in convex regions lead to an increase in reaction rate, while the overall lower temperatures in concave pockets reduce carbon-monoxide consumption. Notably, the reaction progress variable based on product formation remains insensitive to curvature variations, evaluated conditionally on temperature development.

**Figure 5:**
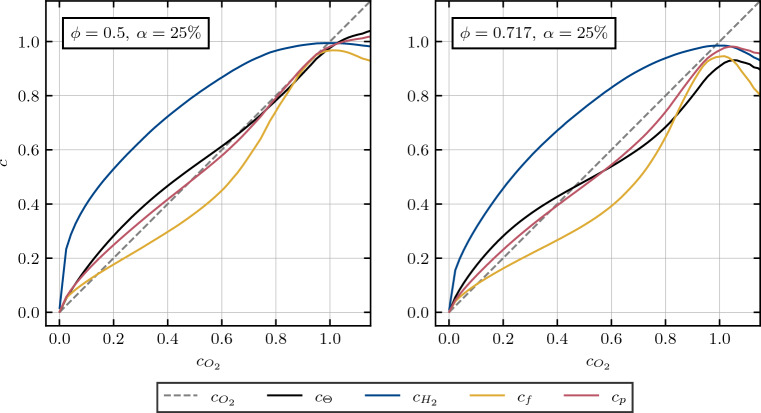
Profiles of mean values of reaction progress variables *c* conditioned upon the reaction progress of oxygen, evaluated for different equivalence ratios $\phi = 0.5$ (left) and $\phi = 0.717$ (right) with a molar carbon-monoxide percentage of $\alpha = 25\%$ in the unburned fuel mixture.

To compare the performance of the product based with the temperature based reaction progress in describing species distribution throughout the flame, both are evaluated conditionally over the oxygen reaction progress in Fig. 4 (right). As anticipated from the observations in Fig. 4 (left), local flame curvature affects temperature and major product development similarly while in general, the product based *c* appears to be slightly more advantageous in describing the species distribution, i.e. it better approximates $c_{O_2}$. To further evaluate these performance differences, two different cases with equivalence ratios $\phi = 0.5$ and $\phi = 0.717$ with a molar carbon-monoxide percentage of $\alpha = 25\%$ in the unburned fuel mixture are assessed in Fig. 5.

It becomes apparent, that the general trend observed in Fig. 4 (right), is also transferable to the other cases shown in Fig.  5. While a comparison between product and temperature based reaction progress variables shows only minor differences in reproducing the oxygen distribution for the low equivalence ratio of $\phi = 0.5$, the advantage of the product based evaluation becomes increasingly apparent with rising equivalence ratios, notable for the $\phi = 0.6$ case in Fig. 4 (right) and the $\phi = 0.717$ case in Fig. 5 (right). Furthermore, it should be noted that the trends of normalized species distributions, as displayed in Figs. 4 and 5, are qualitatively identical for all considered progress variable definitions, regardless on which species (combinations) they are conditionally evaluated on. Hence, these trends are shown in Fig. 5 only for the normalized species profile conditionally on oxygen mass fraction, since the deviations are quantitatively the strongest here and thus best observable in plots.

**Figure 6:**
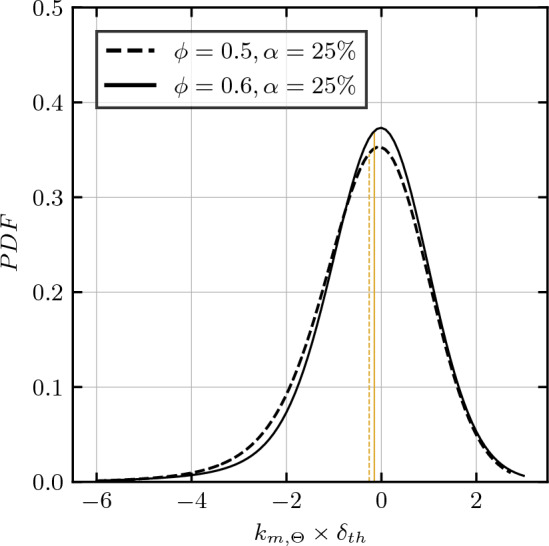
*PDF*s of the normalized curvature of the non-dimensional temperature isosurface $k_{m,\Theta } \times \delta _{th}$, conditionally evaluated for $0.45 \le c_\Theta \le 0.55$. The dashed line represents an equivalence ratio of $\phi = 0.5$, while the solid line represents $\phi = 0.6$. The yellow lines represent the respective mean values. Both cases correspond to a molar fuel composition of $\alpha = 25\%$ carbon-monoxide and a turbulence intensity of $u^\prime / S_L = 4.0$.

Upon inspecting the distribution of convex and concave regions throughout the entire flame domain, the probability density function for the mean curvature is assessed in Fig. 6 for two cases, representing equivalence ratios of $\phi = 0.5$ and $\phi = 0.6$ while both cases correspond to a molar fuel blend of $\alpha = 25\%$ carbon-monoxide. The evaluation is conducted within $0.45 \le c_\Theta \le 0.55$, aligning with the maximum heat release among the considered cases at approximately $c_\Theta \approx 0.5$. Notably, the probability density expresses negative skewness as well as a slight shift of the expected value towards negative values for both considered cases, favoring negative curvatures and therefore signifying the prevalence of concave flame pockets. Existing literature indicates that a flame exhibiting the curvature probability density function shifting toward negative curvature is indicative of the development of intrinsic instabilities, as supported by several studies^44–46^.

**Figure 7:**
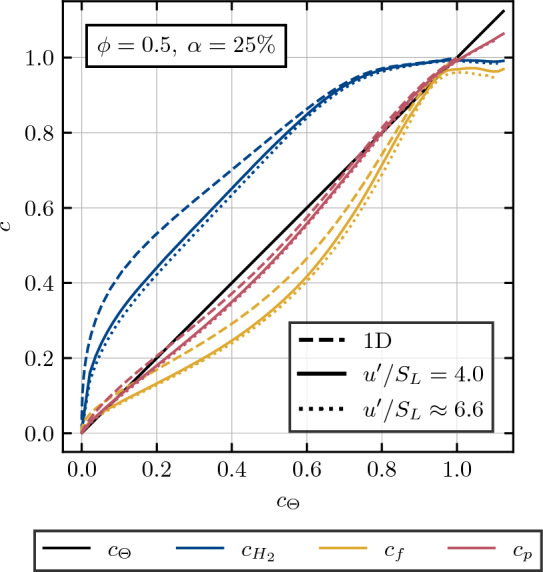
Profiles of mean values of *c* conditioned upon non-dimensional temperature. Dashed lines represent the values corresponding to the 1D laminar case, while solid and dotted lines represent the reaction progress in the turbulent 3D cases with turbulence intensities of $u^\prime / S_L = 4.0$ and $u^\prime / S_L \approx 6.6$ respectively.

When comparing the 1D laminar reaction progress to its counterpart in 3D turbulent simulations for different turbulence intensities, a noticeable shift in their profiles becomes evident in the single-species, mass-fraction based reaction progress variables, as illustrated in Fig. 7. Particularly towards the unburned side and in the intermediate state of the flame ($c_{H_2} < 0.5$), hydrogen displays significantly higher reaction progress in the 1D simulations. This deviation can be attributed to spatial distribution effects unique to 3D simulations, which are further strengthened by the interaction between the turbulent flow field and the flame surface. This uneven spatial distribution is also reflected in the curvature distribution throughout the flame, as can be seen in Fig. 6, where the higher skewness in the negatively curved regions of the turbulent flames indicates the occurrence of localized spatial regions in which the overall reaction rate for hydrogen is significantly decreased. The variation in turbulence intensity, ranging from $u^\prime / S_L = 4.0$ to $u^\prime / S_L = 6.551$, induces an increase in the corresponding Karlovitz number, rising from $Ka = 4.286$ to $Ka = 8.982$. The influence of this turbulence variation is found to be weak within the considered range, aligning well with the findings by Yang et al.^47^.

The product-based reaction progress variable exhibits a consistent behavior between both 1D and 3D simulations, with minimal sensitivity to spatial distribution effects and turbulence variations. However, the product-based reaction progress variable assumes values greater than unity in the super-adiabatic zones. This can be problematic for some mean reaction rate closures which depend for example on $\tilde{c}(1-\tilde{c})$^48^ where $\tilde{c}$ is the Favre-filtered value of reaction progress variable. A modelling approach which depends on $|\nabla \tilde{c}|$^49,50^ does not necessarily need the boundedness of $\tilde{c}$ within 0.0 and 1.0 in the framework of stretched flamelets where the chemistry tabulation accounts for superadiabatic values of reaction progress variable based on a combination of major product species. It is worth noting that the present results show that $c_{H_2}$ performs the best from the point of view of the boundedness between 0.0 and 1.0 despite showing sensitivity to preferential diffusion effects especially in the presence of flame curvature. Thus, a reaction progress variable based on $c_{H_2}$ should be preferred if the boundedness of *c* is critical for the model approach.

## Conclusion

In this study, direct numerical simulation data with detailed chemistry is utilized to investigate pure hydrogen/air and syngas/air flames in both 1D unstretched laminar and turbulent 3D cases. The analysis is focused on the impact of different mixtures, turbulence intensities and flame topologies on the reaction progress of various chemical species and combinations of species.

It has been found that a reaction progress variable based on oxygen does not adequately capture the preferential diffusion effects on the normalized burning rate per unit flame surface area and shows an opposing trend in comparison to the normalized burning rate per unit flame surface area obtained for hydrogen and product mass fraction based reaction progress variables.

While evaluations based on other single-species reaction progress variables, such as the hydrogen-based reaction progress, effectively describe the elevated normalized burning rate per unit area of the flame, they exhibit high sensitivity to preferential diffusion effects, particularly depending on the local flame topology. This increased sensitivity is also evident in the reaction progress based on a linear combination of mass fractions of all fuel species, notably in response to variations in the fuel composition.

However, a reaction progress variable based on all major product species demonstrates the ability to accurately represent the temperature evolution within the flame across different mixtures, turbulence intensities, and flame topologies while also being able to effectively capture the preferential diffusion effects on the normalized burning rate per unit flame surface area, suggesting that this choice of reaction progress variable is well-suited for future subgrid modeling approaches for turbulent syngas-air premixed flames. Yet it has to be taken into account that, as a result of preferential diffusion, this choice tends to assume values greater than 1.0 in the super-adiabatic temperature regions. This presents challenges for Arrhenius-type models, such as Artificial Thickened Flame models^51^, which assume Favre-filtered reaction progress variables that do not surpass 1.0 in the reaction rate source term in the reaction progress variable transport equation $\overline{\dot{\omega }}_c \propto \overline{\rho } \left( 1 - \tilde{c} \right) \exp {\left[ -T_{act} /\tilde{T} \right] }$ where $T_{act}$ is the activation temperature. Moreover, some of the closures of $\overline{\dot{\omega }}_c$ involve $\tilde{c}(1-\tilde{c})$^48^ and a value of $\tilde{c}>1$ is expected to yield unphysical trends for these closures. For such models, a hydrogen-based reaction progress variable ($c_{H_2}$) is preferred, albeit its sensitivity towards local flame topology, because it is well bound between 0.0 and 1.0 and shows reduced sensitivity to fuel composition changes compared to variables based on a primary fuel species ($c_f$). However, for models focusing on the gradient of the reaction progress variable ($|\nabla \tilde{c}|$), such as the Flame Surface Density modelling approaches^49,50^, using a combination of major product species effectively captures the chemical reaction progress within the flame front, provided a stretched flamelet approach is adopted where the chemistry tabulation accounts for superadiabatic values of reaction progress variable based on a combination of major product species. The information based on normalized mass fractions of individual species and the combination of fuel and product species can form the basis of the set that can be used to optimize the weights, forming an optimized reaction progress variable based on an automated method^14–18^.

## Data Availability

The data that support the findings of this study are available from the corresponding author upon reasonable request.
